# Efficacy and Safety of Isatuximab Combination Therapy in Multiple Myeloma: A Meta-Analysis of Randomized Controlled Trials

**DOI:** 10.3390/cancers17213494

**Published:** 2025-10-30

**Authors:** Chi Wang, Zhengyang Xu, Meilin Jiang, Yuzhe Chen, Yu Lan

**Affiliations:** State Key Laboratory of Experimental Hematology, Key Laboratory for Regenerative Medicine of Ministry of Education, Institute of Hematology, School of Medicine, Jinan University, Guangzhou 510632, China

**Keywords:** isatuximab, multiple myeloma, randomized controlled trials, efficacy, safety, meta-analysis

## Abstract

**Simple Summary:**

Multiple myeloma is a type of blood cancer that often returns after initial treatment, creating a need for more effective therapies. Isatuximab is a newer antibody drug that helps the patient’s own immune system attack the cancer cells. While several clinical trials have shown that adding isatuximab to standard treatments can be beneficial, the overall picture from these individual studies can be unclear. Our research combined the results of all relevant high-quality trials to provide a definitive answer. We found that treatment regimens containing isatuximab significantly delay cancer progression and lead to deeper responses in both newly diagnosed and relapsed patients, with side effects that are generally manageable. This analysis helps confirm the value of isatuximab, giving doctors and patients greater confidence in using it as part of their treatment strategy.

**Abstract:**

Background: This meta-analysis evaluates the efficacy and safety of isatuximab, an anti-CD38 monoclonal antibody, in combination regimens for newly diagnosed (NDMM) and relapsed/refractory multiple myeloma (RRMM). Methods: We systematically searched major databases for randomized controlled trials (RCTs) comparing isatuximab-based therapy with standard regimens up to September 2025. Efficacy and safety analyses were performed separately for NDMM and RRMM populations using random-effects models. Efficacy outcomes included progression-free survival (PFS), overall survival (OS), overall response rate (ORR), very good partial response (VGPR) or better, and minimal residual disease (MRD) negativity rate. Safety was assessed by grade ≥ 3 adverse events. Results: In NDMM patients, isatuximab significantly improved PFS (HR = 0.66, 95% CI: 0.52–0.84, *p* = 0.001) and MRD negativity rates (RR = 1.28, 95% CI: 1.13–1.45, *p* < 0.001), but not OS (HR = 1.01, *p* = 0.937), ORR (RR = 1.02, *p* = 0.49), or VGPR or better (RR = 1.10, *p* = 0.13). In RRMM patients, isatuximab significantly improved PFS (HR = 0.61, 95% CI: 0.50–0.74, *p* < 0.001) and showed strong trends favoring OS (HR = 0.81, 95% CI: 0.65–1.00, *p* = 0.051) and ORR (RR = 1.30, 95% CI: 0.79–2.16, *p* = 0.303), while significantly increasing MRD negativity (RR = 4.37, 95% CI: 0.60–31.68, *p* = 0.144). A trend toward improved OS was observed in RRMM (HR = 0.81, *p* = 0.051). In NDMM, PFS benefit was significant for standard-risk but not high-risk cytogenetics. Safety analysis showed an increased risk of grade ≥ 3 adverse events RRMM (RR = 1.18, *p* < 0.001) but not in NDMM (RR = 1.08, *p* = 0.064), primarily driven by neutropenia (NDMM RR = 1.96, *p* = 0.003; RRMM RR = 1.77, *p* = 0.039) and pneumonia in NDMM (RR = 1.80, *p* = 0.001). Conclusion: Isatuximab-based regimens significantly improve PFS and depth of response with a manageable safety profile, supporting its use across MM settings, though efficacy in NDMM may vary by cytogenetic risk.

## 1. Introduction

Multiple myeloma (MM) is a prevalent hematologic malignancy, accounting for approximately 1% of all cancers, and is characterized by the clonal proliferation of plasma cells in the bone marrow [1,2]. Despite therapeutic advances, including proteasome inhibitors and immunomodulatory drugs, it remains largely incurable, with most patients eventually relapsing [3,4,5,6]. This reality underscores the need for new treatments that work through different mechanisms.

Targeting the CD38 protein on myeloma cells with monoclonal antibodies has proven to be a successful strategy [7]. The first approved anti-CD38 antibody, daratumumab, improved survival for patients with both newly diagnosed and relapsed/refractory disease, confirming CD38′s value as a target [8,9,10]. Isatuximab is a distinct anti-CD38 monoclonal antibody that binds a unique epitope and is reported to induce tumor cell death through multiple mechanisms, including direct apoptosis, antibody-dependent cellular cytotoxicity, phagocytosis, and inhibition of CD38 enzymatic activity [11,12]. Its application has expanded from the relapsed/refractory setting to include frontline treatment for transplant-ineligible newly diagnosed multiple myeloma (NDMM) [13,14]. Preclinical studies suggest that its unique mechanism of action may also modulate the immunosuppressive bone marrow microenvironment, potentially offering advantages in certain clinical contexts [15].

Recent phase III trials, such as ICARIA-MM and IKEMA, with updated follow-up data, have established the efficacy of isatuximab combined with standard regimens (e.g., pomalidomide-dexamethasone or carfilzomib-dexamethasone) in RRMM [16,17,18]. Furthermore, in NDMM, trials including IMROZ and GMMG-HD7 have reported improved outcomes with isatuximab-based quadruplet regimens [19,20,21]. However, the results across individual studies vary, and the overall benefit-risk profile of isatuximab across different MM settings, patient populations, and in comparison with other therapeutic options requires a more precise and comprehensive elucidation. While previous meta-analyses have explored anti-CD38 therapies [22], the body of evidence for isatuximab has grown substantially, necessitating an updated synthesis to provide robust, quantitative conclusions on its efficacy and safety.

Therefore, we conducted this meta-analysis to synthesize evidence from all relevant randomized controlled trials (RCTs). We aimed to definitively evaluate the efficacy and safety of isatuximab-based regimens compared to standard therapies for MM.

## 2. Materials and Methods

### 2.1. Literature Search

This meta-analysis was conducted in accordance with Preferred Reporting Items for Systematic Reviews and Meta-Analyses (PRISMA) guidelines (Table S1) [23]. The study was registered in the International Platform of Registered Systematic Review and Meta-analysis Protocols (registration number: INPLASY2025100027). We conducted a systematic literature search of electronic databases, including PubMed, EMBASE, Cochrane Central Register of Controlled Trials, and Web of Science, from inception to September 2025. The search strategy incorporated Medical Subject Headings (MeSH) terms and free-text words related to “isatuximab”, “multiple myeloma”, “randomized controlled trial”, and their variations. The complete search strategy for PubMed is provided in Table S2. In addition to database searches, we manually searched the reference lists of relevant review articles to identify any potentially eligible trials that might have been missed. Two investigators independently screened titles and abstracts of identified records, followed by full-text assessment of potentially eligible studies. Discrepancies were resolved through discussion or consultation with a third reviewer.

### 2.2. Inclusion and Exclusion Criteria

Studies were included if they met the following criteria: (1) RCTs; (2) included adult patients (≥18 years) with NDMM/RRMM; (3) compared isatuximab-containing regimens with standard therapies; (4) reported at least one efficacy outcome (PFS, OS, ORR, VGPR or better, MRD negativity) or safety outcome (grade ≥ 3 adverse events).

Exclusion criteria included: (1) non-randomized studies, case reports, reviews, and editorials; (2) studies with overlapping patient populations; (3) studies published in languages other than English; (4) ongoing trials without available results.

### 2.3. Data Extraction and Quality Assessment

Two reviewers independently extracted data using a pre-designed electronic form. The extracted data included (1) study characteristics: first author, publication year, study design, and sample size; (2) participant characteristics: age, disease status, cytogenetic risk; (3) intervention details: regimen, dosage; and (4) outcome data for all pre-specified efficacy and safety endpoints. For time-to-event outcomes (PFS, OS), we extracted hazard ratios (HRs) with 95% confidence intervals (CIs). For dichotomous outcomes, we extracted event counts and total numbers in each group.

The methodological quality of the included studies was independently assessed by two reviewers using the Cochrane Risk of Bias tool, evaluating key domains such as randomization, blinding, and outcome reporting [24]. For the ‘Other bias’ domain, as per Cochrane guidelines, we assessed whether each study was free of other problems that could put it at a high risk of bias. This included an assessment of potential bias related to the study’s funding source and the role of the funder in study design, conduct, analysis, or reporting, as disclosed in the publications. Disagreements were resolved through discussion or by consulting a third reviewer.

### 2.4. Statistical Analysis

All statistical syntheses were conducted with RevMan 5.4 and Stata 14. Given the anticipated clinical heterogeneity across trials in terms of patient populations and treatment regimens, all analyses were performed using random-effects models. Pooled HRs for time-to-event outcomes (PFS and OS) and risk ratios (RRs) for dichotomous outcomes (ORR, MRD negativity, VGPR or better, and adverse events) were estimated, each presented with their 95% CIs. To address fundamental differences in disease biology and treatment goals, all efficacy and safety analyses were conducted separately for NDMM and RRMM populations. The degree of statistical heterogeneity was assessed using the I^2^ statistic [25,26].

Sensitivity and publication bias analyses were pre-specified to assess robustness. However, the limited number of studies included in each meta-analysis (often only 2–3 per comparison) precluded the use of these methods. As per methodological guidelines (e.g., the Cochrane Handbook for Systematic Reviews of Interventions), sensitivity analysis by study removal is not recommended with so few studies, as it yields unstable estimates [27]. Similarly, tests for publication bias (e.g., funnel plots, Egger’s test) are unreliable with fewer than ten studies and were therefore omitted [28]. Instead, the robustness of the findings was evaluated through pre-specified subgroup analyses and careful inspection of forest plots, as detailed in the Results. All statistical tests were two-sided, with a significance threshold of *p* < 0.05.

## 3. Results

### 3.1. Study Selection and Characteristics

**Figure 1:**
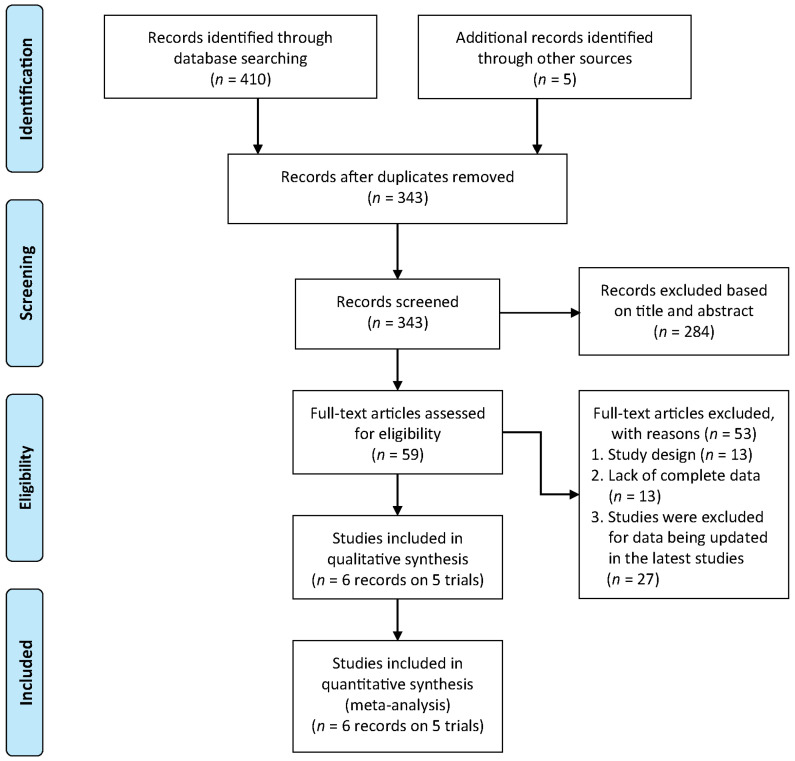
Flow chart of PRISMA.

The systematic literature search and selection process are summarized in Figure 1. Of the 415 initially identified records, 6 articles (reporting on 5 RCTs) met the eligibility criteria and were included in the meta-analysis after a systematic screening process [16,18,19,20,21,29], which excluded records primarily for outdated data, incompatible study design, or insufficient reporting. These 5 RCTs encompassed a total of 2017 patients. Among these, three trials focused on NDMM patients (*n* = 1408), and two enrolled RRMM patients (*n* = 609). The regimens included are isatuximab combined with pomalidomide-dexamethasone (Isa-Pd), carfilzomib-dexamethasone (Isa-Kd), lenalidomide-dexamethasone (Isa-Rd), and bortezomib-based triplets. The median follow-up duration ranged from 20 to 60 months across studies. The baseline characteristics of the included studies are summarized in Table 1.

### 3.2. Efficacy Outcomes

#### 3.2.1. PFS and OS

**Figure 2:**
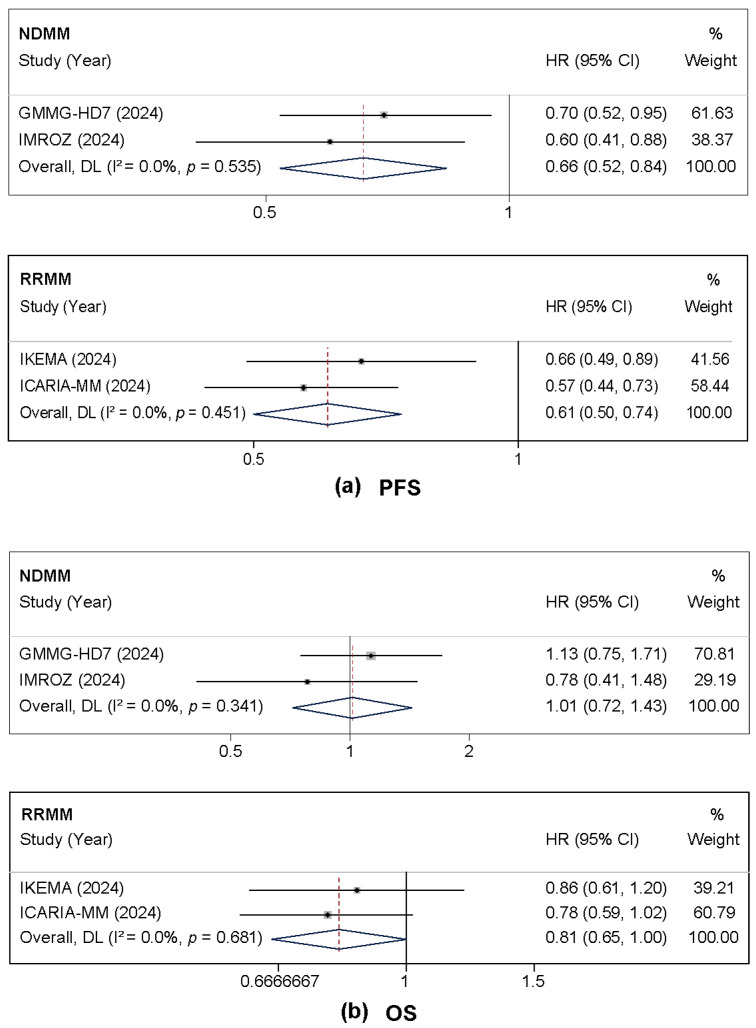
Forest plots of hazard ratios (HRs) comparing isatuximab-based regimens to control. All analyses used the random-effects model. (**a**) Progression-free survival (PFS). (**b**) Overall survival (OS). For each outcome, the forest plot for patients with newly diagnosed multiple myeloma (NDMM) is shown above the plot for those with relapsed/refractory disease (RRMM) [16,18,20,21].

Isatuximab-based regimens demonstrated significant improvement in PFS across both NDMM and RRMM populations. In the NDMM subgroup, isatuximab reduced the risk of disease progression or death by 34% (HR = 0.66, 95% CI: 0.52–0.84, *p* = 0.001) with no heterogeneity observed (I^2^ = 0%). Similarly, in the RRMM subgroup, the risk was reduced by 39% (HR = 0.61, 95% CI: 0.50–0.74, *p* < 0.001), also with no heterogeneity (I^2^ = 0%) (Figure 2a).

For OS, distinct patterns were observed between populations. In NDMM patients, no significant OS benefit was observed with isatuximab (HR = 1.01, 95% CI: 0.72–1.43, *p* = 0.937), likely reflecting the immaturity of survival data and the impact of subsequent therapies in this population. Conversely, the RRMM subgroup demonstrated a clinically meaningful trend toward improved survival (HR = 0.81, 95% CI: 0.65–1.00, *p* = 0.051) (Figure 2b).

#### 3.2.2. Treatment Response and MRD Negativity

**Figure 3:**
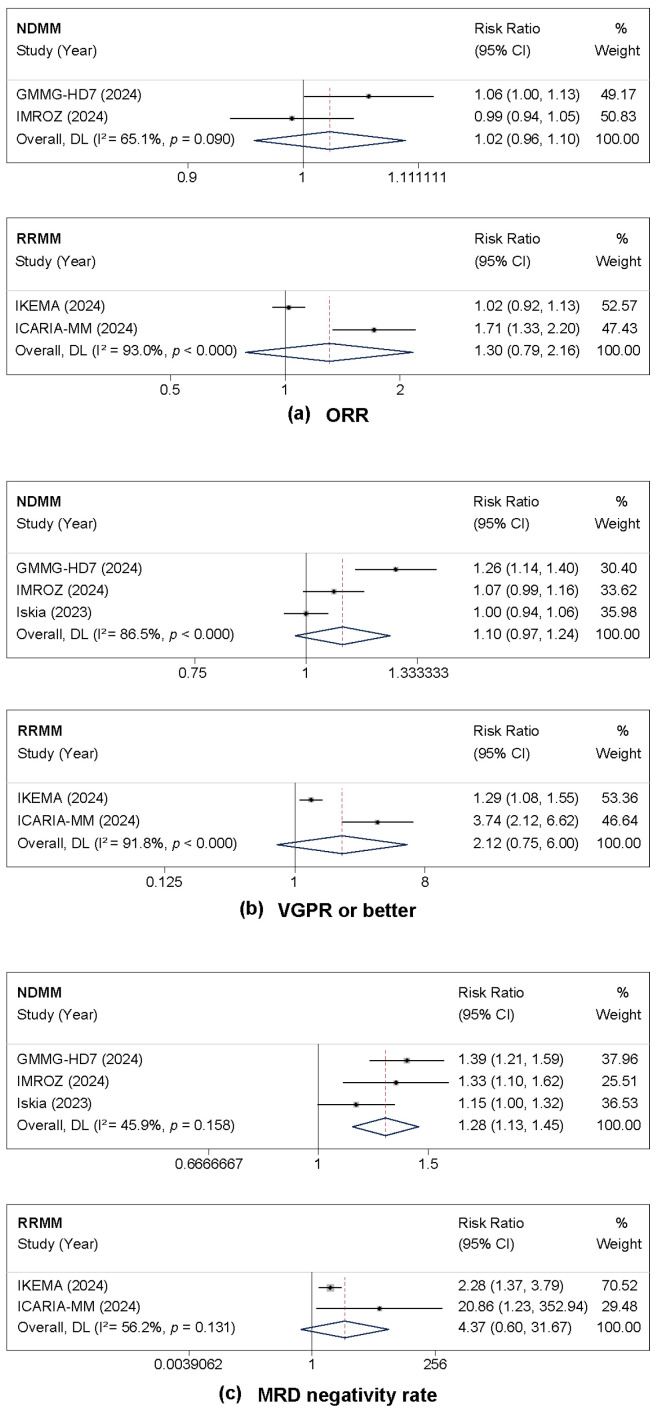
Forest plots of risk ratios (RRs) comparing isatuximab-based regimens to control. All analyses used the random-effects model. (**a**) Overall response rate (ORR). (**b**) Very good partial response (VGPR) or better. (**c**) Minimal residual disease (MRD) negativity rate. For each outcome, the forest plot for patients with newly diagnosed multiple myeloma (NDMM) is shown above the plot for those with relapsed/refractory disease (RRMM) [16,18,19,20,21,29].

The analysis of response rates revealed that isatuximab-based therapy was associated with improved depth of response across multiple endpoints. For the ORR, no significant improvement was observed in either the NDMM (RR = 1.02, 95% CI: 0.96–1.10, *p* = 0.490) or RRMM subgroups (RR = 1.30, 95% CI: 0.79–2.16, *p* = 0.303). Substantial heterogeneity was noted in the RRMM analysis (I^2^ = 93.0%) (Figure 3a).

The rate of patients achieving VGPR or better showed a non-significant trend favoring isatuximab in NDMM (RR = 1.10, 95% CI: 0.97–1.24, *p* = 0.130) and a more pronounced but also non-significant effect in RRMM (RR = 2.13, 95% CI: 0.75–6.00, *p* = 0.155). Considerable heterogeneity was noted for this outcome in both subgroups (I^2^ = 86.5% for NDMM and 91.8% for RRMM) (Figure 3b).

A key finding was the significant improvement in MRD negativity rates with isatuximab treatment in NDMM patients, demonstrating a 28% increase (RR = 1.28, 95% CI: 1.13–1.45, *p* < 0.001), with moderate heterogeneity (I^2^ = 45.9%). In RRMM patients, the point estimate indicated a more than four-fold increase in MRD negativity (RR = 4.37, 95% CI: 0.60–31.68, *p* = 0.144), although the confidence interval was wide and the result was not statistically significant, with moderate heterogeneity (I^2^ = 56.2%) (Figure 3c).

### 3.3. Safety Outcomes

#### 3.3.1. Grade 3 or 4 Adverse Events and Fatal Adverse Events

**Figure 4:**
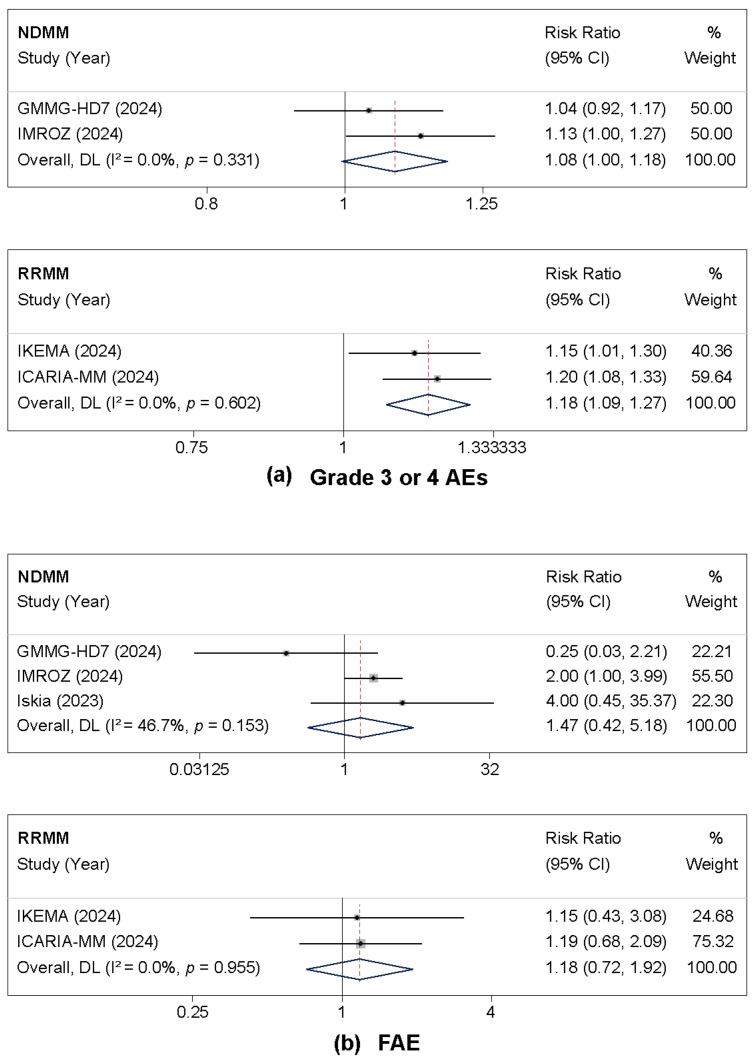
Forest plots of risk ratios (RRs) comparing isatuximab-based regimens to control for grade ≥ 3 adverse events. All analyses used the random-effects model. (**a**) Grade 3 or 4 adverse events. (**b**) Fatal adverse event (FAE). For each outcome, the forest plot for patients with newly diagnosed multiple myeloma (NDMM) is shown above the plot for those with relapsed/refractory disease (RRMM) [16,18,19,20,21,29].

The safety analysis encompassed 2017 patients from the included trials. The incidence of grade 3 or 4 adverse events was significantly higher in the isatuximab group for RRMM patients (RR = 1.18, 95% CI: 1.09–1.27, *p* < 0.001), with low heterogeneity (I^2^ = 0%). In NDMM patients, a non-significant trend towards increased risk was observed (RR = 1.08, 95% CI: 1.00–1.18, *p* = 0.064) (Figure 4a). Regarding fatal adverse events, while there was a numerical increase in the isatuximab group in both NDMM (RR = 1.47, 95% CI: 0.42–5.18, *p* = 0.551) and RRMM (RR = 1.18, 95% CI: 0.72–1.92, *p* = 0.514), neither reached statistical significance (Figure 4b).

#### 3.3.2. Hematologic Adverse Events

Isatuximab treatment was associated with a substantially increased risk of grade 3 or 4 neutropenia in both NDMM (RR = 1.96, 95% CI: 1.26–3.07, *p* = 0.003) and RRMM subgroups (RR = 1.77, 95% CI: 1.03–3.05, *p* = 0.039), with substantial heterogeneity in the NDMM analysis (I^2^ = 81.2%). In contrast, the risks of grade 3 or 4 thrombocytopenia (NDMM: RR = 1.08, 95% CI: 0.85–1.37, *p* = 0.552; RRMM: RR = 1.21, 95% CI: 0.87–1.67, *p* = 0.260) and anemia (NDMM: RR = 0.90, 95% CI: 0.55–1.48, *p* = 0.680; RRMM: RR = 2.06, 95% CI: 0.39–11.04, *p* = 0.399) were not significantly different between treatment groups (Supplementary Figure S1).

#### 3.3.3. Non-Hematologic Adverse Events

The analysis of non-hematologic toxicities revealed a significantly increased risk of grade 3 or 4 pneumonia with isatuximab treatment in NDMM patients (RR = 1.80, 95% CI: 1.27–2.55, *p* = 0.001), with low heterogeneity (I^2^ = 0%). This risk was not significantly elevated in the RRMM subgroup (RR = 1.22, 95% CI: 0.88–1.68, *p* = 0.227). No significant differences were observed for grade 3 or 4 diarrhea in the RRMM subgroup (RR = 1.26, 95% CI: 0.42–3.82, *p* = 0.678); data were insufficient for meta-analysis in NDMM. For fatigue, the RRMM subgroup demonstrated a significantly increased risk (RR = 9.02, 95% CI: 1.72–47.40, *p* = 0.009), while data in NDMM were insufficient for meta-analysis (Supplementary Figure S2).

### 3.4. Subgroup Analysis by Cytogenetic Risk and Disease Status

A pre-specified subgroup analysis of PFS was performed to evaluate the efficacy of isatuximab according to cytogenetic risk, stratified by disease status (NDMM vs. RRMM).

**Figure 5:**
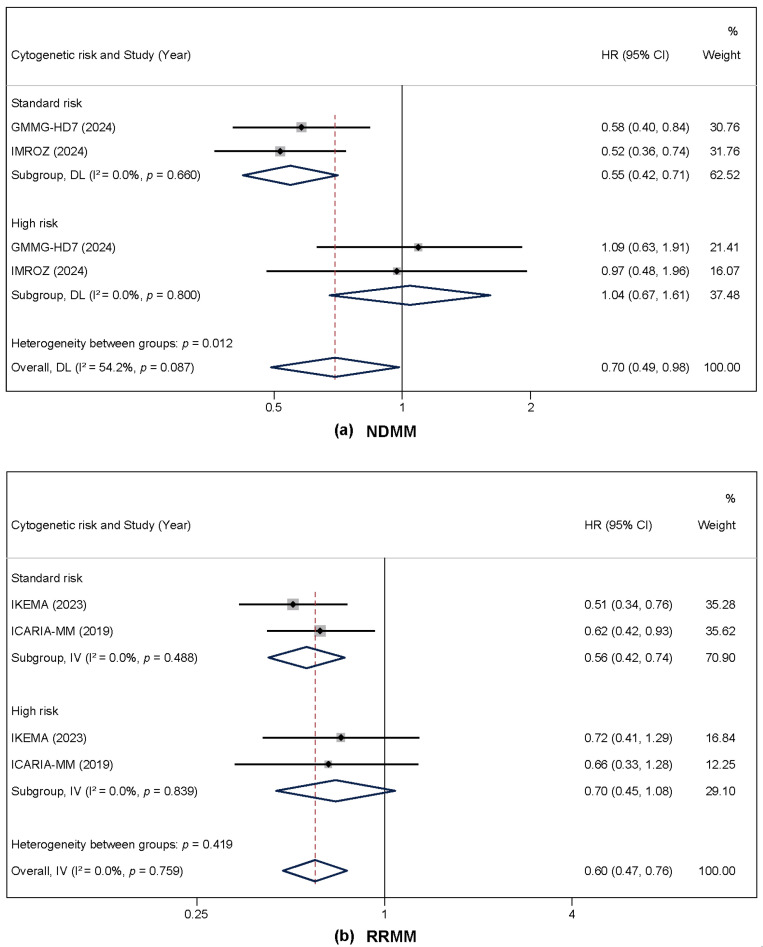
Forest plots of hazard ratios (HRs) for progression-free survival (PFS) from subgroup analysis by cytogenetic risk and disease status comparing isatuximab-based regimens to the control arm. (**a**) Newly diagnosed multiple myeloma (NDMM). (**b**) Relapsed/refractory multiple myeloma (RRMM). The random-effects model was used [16,18,20,21].

In patients with NDMM, the treatment effect of isatuximab demonstrated significant interaction with cytogenetic risk (*p* for interaction = 0.012). A substantial and statistically significant PFS benefit was observed in the standard-risk subgroup (HR = 0.55; 95% CI, 0.42–0.71; *p* < 0.001), indicating a 45% reduction in the risk of progression or death. Conversely, no significant benefit was detected in the high-risk NDMM subgroup (HR = 1.04; 95% CI, 0.68–1.61; *p* = 0.851). The overall pooled estimate for NDMM was HR = 0.70 (95% CI, 0.49–0.98; *p* = 0.04), with moderate heterogeneity (I^2^ = 54.2%) attributable to the divergent effects between risk subgroups (Figure 5a).

In patients with RRMM, the PFS benefit of isatuximab was consistent across cytogenetic risk subgroups, with no significant interaction observed (*p* for interaction = 0.419). A significant improvement in PFS was demonstrated in the standard-risk subgroup (HR = 0.56; 95% CI, 0.42–0.74; *p* < 0.001). In the high-risk RRMM subgroup, a trend towards improved PFS was observed (HR = 0.70; 95% CI, 0.45–1.08; *p* = 0.107), although this did not reach statistical significance. The overall pooled estimate for RRMM was HR = 0.60 (95% CI, 0.47–0.76; *p* < 0.001), with no heterogeneity (I^2^ = 0%) (Figure 5b).

### 3.5. Risk of Bias Assessment

**Figure 6:**
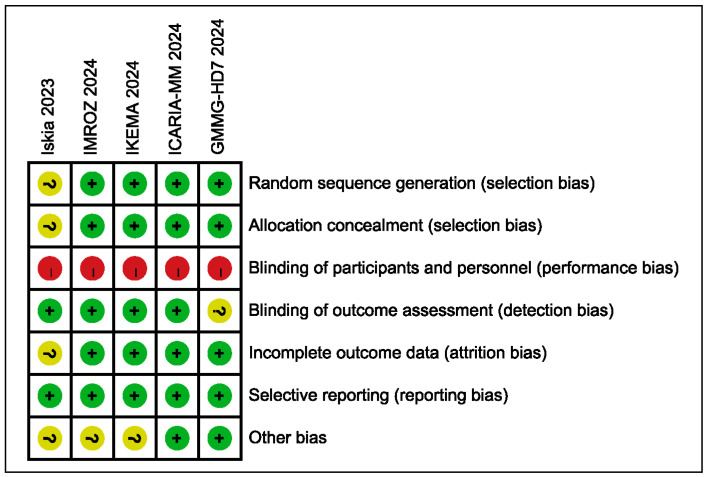
Risk of bias summary of the included studies. Green (+, low risk), yellow (?, some concerns), and red (−, high risk) represent the judgments for each risk of bias item [16,18,19,20,21,29].

All trials demonstrated low risk of bias concerning random sequence generation and allocation concealment, ensuring the reliability of the randomization process. The primary concern across all studies was performance bias due to their open-label nature. However, this was substantially mitigated for the primary efficacy endpoints in four of the five trials (IKEMA, ICARIA-MM, IMROZ, and IsKia) through the use of blinded Independent Review Committees or central laboratory assessments, thus reducing detection bias. While the heterogeneity in patient populations (newly diagnosed versus relapsed/refractory) and specific treatment regimens exists, the consistent demonstration of a significant PFS benefit and deeper responses with isatuximab across these well-conducted trials strengthens the validity and generalizability of the collective findings. Consequently, despite the open-label design, the results are considered reliable for informing clinical practice (Figure 6).

## 4. Discussion

This meta-analysis consolidates evidence from randomized controlled trials to demonstrate that isatuximab-based regimens significantly enhance PFS and deepen treatment responses, as measured by MRD negativity, in both NDMM/RRMM, though the magnitude of benefit varies between these distinct populations. The observed safety profile, while indicating an increased risk for specific hematological and infectious adverse events, remains manageable and consistent with the mechanisms of anti-CD38 therapy [30]. These findings not only affirm the value of isatuximab in the therapeutic arsenal for multiple myeloma but also invite a deeper exploration of its position within the broader context of anti-CD38 treatments.

The significant PFS benefit observed with isatuximab in both NDMM (HR = 0.66) and RRMM (HR = 0.61) aligns with the established efficacy of the anti-CD38 class. As summarized in Table S3, the magnitude of PFS benefit with isatuximab is comparable to that reported for daratumumab in their respective pivotal trials [31,32]. A descriptive, indirect comparison of key outcomes is provided in Table S3, visually consolidating the evidence for this potent class effect. It is important to note, however, that in the NDMM population, the daratumumab-based regimen demonstrated a statistically significant PFS benefit in patients with high-risk cytogenetics but not in those with standard-risk disease. This convergence of efficacy highlights the transformative impact of targeting CD38. However, indirect comparisons across different trial platforms should be interpreted with caution due to differences in patient populations, backbone regimens, and follow-up duration. Furthermore, a critical distinction emerges in the subgroup analysis by cytogenetic risk. Our analysis suggests a consistent PFS benefit for isatuximab in RRMM patients regardless of cytogenetic risk, whereas in NDMM, the benefit appears more pronounced in standard-risk patients. This nuanced finding partially warrants further investigation, as it may be influenced by variations in trial populations, backbone regimens, or other clinical factors.

A paramount finding of this study is the significant improvement in MRD negativity rates with isatuximab-based therapy in NDMM (RR = 1.28), and a very substantial increase in RRMM (RR = 4.37), albeit with a wide confidence interval in the latter. This ability to deeply suppress the disease is a hallmark of anti-CD38 therapy, with both isatuximab and daratumumab demonstrating significantly higher MRD negativity rates compared to their respective control arms (Table S3). Achieving MRD negativity is increasingly recognized as a robust surrogate for long-term clinical outcomes and is considered a cornerstone in the strategy to achieve a “functional cure” for multiple myeloma [33,34,35]. The depth of response elicited by isatuximab, as captured by MRD, underscores its potency and may ultimately translate into sustained survival advantages with extended follow-up. This is corroborated by the recent expert review by Richardson et al., which emphasizes that isatuximab-based quadruplet regimens in NDMM are achieving some of the highest MRD negativity rates observed, linking this depth of response directly to improved PFS [36].

The trend towards an OS benefit in the RRMM subgroup (HR = 0.81, *p* = 0.051) is clinically suggestive and finds support in updated results from pivotal trials. For instance, the long-term follow-up of the IKEMA trial demonstrated a significant OS advantage for isatuximab-carfilzomib-dexamethasone over standard therapy [18]. The current absence of a statistically significant OS benefit in our NDMM analysis is not unexpected; it likely reflects the immaturity of survival data in this population and the confounding effect of numerous effective subsequent therapies, which can obscure the impact of first-line treatment on OS.

Regarding safety, the profile of isatuximab is characterized by an increased risk of grade ≥ 3 neutropenia and infections such as pneumonia, effects consistent with the immunomodulatory actions of anti-CD38 antibodies [22,37]. As anticipated for this drug class and detailed in Table S3, both isatuximab and daratumumab are associated with a pronounced increase in the risk of grade ≥ 3 neutropenia and a higher incidence of pneumonia compared to standard regimens, confirming a shared and manageable safety consideration. The striking increase in severe fatigue observed in the RRMM population (RR = 9.02) is a notable finding that merits clinical attention and further investigation into its underlying mechanisms. It is also associated with infusion reactions, which occur in about a third to half of patients, primarily during the first dose. Notably, the risks of grade ≥ 3 thrombocytopenia and anemia were not significantly elevated compared to controls. This hematological toxicity profile is manageable in clinical practice with vigilant monitoring, growth factor support, and antimicrobial prophylaxis. However, emerging data support the feasibility of rapid infusion (over 30 min) after the first two tolerated doses, which has been shown to improve patient convenience and clinic efficiency without increased reactions. A comprehensive safety evaluation by Karimbanathottathil et al., which integrated data from clinical trials and real-world pharmacovigilance, confirmed these signals while concluding that isatuximab maintains a manageable safety profile [38].

When contextualizing isatuximab within the anti-CD38 class, it is pertinent to consider the consolidated data presented in Table S3. While both daratumumab and isatuximab target CD38 and show broadly similar efficacy and safety profiles at a class level, isatuximab binds a distinct epitope and is reported to have stronger direct pro-apoptotic and immunomodulatory effects, including unique inhibition of CD38 enzymatic activity [14]. Whether these mechanistic differences translate into tangible variations in clinical efficacy across specific patient subgroups, such as those with high-risk cytogenetics, remains a subject of ongoing research. Furthermore, administration logistics are evolving; while subcutaneous daratumumab is established in practice, subcutaneous formulations of isatuximab via an on-body delivery system are showing promising results in clinical trials, potentially enhancing patient convenience and tolerability in the future [39,40].

Several limitations inherent in this meta-analysis must be acknowledged. The number of included RCTs is modest, which constrained our ability to perform robust sensitivity analyses or formally assess publication bias for all outcomes. Significant heterogeneity was observed for several efficacy endpoints (e.g., ORR, VGPR), likely attributable to clinical diversity across the trials, including differences in combination regimens, lines of therapy, and patient risk profiles. Furthermore, the OS data, particularly for NDMM, remains immature. Finally, by restricting inclusion to RCTs, we may have excluded real-world evidence that captures the application of these regimens in broader, more diverse clinical practice settings. Nevertheless, a recent real-world study from China on the Isa-Pd regimen in RRMM reported an overall response rate of 82.6% [41], which aligns with and even exceeds the efficacy observed in the pivotal ICARIA-MM trial, thus supporting the generalizability of the trial results.

## 5. Conclusions

In conclusion, this meta-analysis provides consolidated, high-quality evidence that isatuximab significantly improves key efficacy outcomes, including PFS and MRD negativity, with a predictable and manageable safety profile. The efficacy and safety profiles differ between NDMM and RRMM populations, underscoring the importance of considering disease setting in treatment decisions. These findings firmly support the integration of isatuximab-based combinations into standard treatment paradigms for both NDMM and RRMM. Future research should prioritize long-term follow-up to mature overall survival data, biomarker-driven studies to identify patient subsets that derive the maximum benefit, and direct comparative studies to further elucidate the nuanced differences between anti-CD38 monoclonal antibodies.

## Figures and Tables

**Table 1:** The baseline characteristics of studies included in the meta-analysis.

Study	Year ^&^	Phase	Population	Patients		Median Age (Year)		Intervention		Follow-Up ^#^ (Months)	Primary End-Point
				E	C	E	C	E	C		
GMMG-HD7 [19,20]	2024	III	NDMM	331	329	59 (54–64)	60 (54–65)	Isa-RVd	RVd	48	MRD negative rate
IMROZ [21]	2024	III	NDMM	265	181	72 (60–80)	72 (55–80)	Isa-VRd	VRd	59.7	PFS
IKEMA [18]	2024	III	RRMM	179	123	65 (55–77)	63 (57–70)	Isa-Kd	Kd	56.6	PFS
ICARIA-MM [16]	2024	III	RRMM	154	153	68 (60–74)	66 (59–71)	Isa-Pd	Pd	52.4	PFS
Iskia * [29]	2023	III	NDMM	151	151	61	60	Isa-KRd	KRd	20	MRD negative rate

* Only conference abstracts are available for Iskia study; ^&^ Latest publication year; ^#^ median. E: Experimental arm; C: Control arm; Isa: isatuximab; V: bortezomib; R: lenalidomide; d: dexamethasone; K: carfilzomib; P: pomalidomide; MRD: minimal residual disease.

## Supplementary Materials

The following supporting information can be downloaded at: https://www.mdpi.com/article/10.3390/cancers17213494/s1. Figure S1: Forest plots of risk ratios (RRs) for hematologic adverse events comparing isatuximab-based regimens to the control arm. (**a**) Grade 3 or 4 neutropenia. (**b**) Grade 3 or 4 thrombocytopenia. (**c**) Grade 3 or 4 anemia. Figure S2: Forest plots of risk ratios (RRs) for non-hematologic adverse events comparing isatuximab-based regimens to the control arm. (**a**) Grade 3 or 4 pneumonia. (**b**) Grade 3 or 4 diarrhea. (**c**) Grade 3 or 4 fatigue. Table S1: PRISMA 2020 Checklist; Table S2: Search strategy; Table S3: Indirect Comparison of Key Efficacy and Safety Outcomes for Anti-CD38 Antibodies in Multiple Myeloma.

## References

[1] Rajkumar S.V. (2024). Multiple myeloma: 2024 update on diagnosis, risk-stratification, and management. Am. J. Hematol..

[2] Malard F., Neri P., Bahlis N.J., Terpos E., Moukalled N., Hungria V.T.M., Manier S., Mohty M. (2024). Multiple myeloma. Nat. Rev. Dis. Primers..

[3] Bird S., Pawlyn C. (2023). IMiD resistance in multiple myeloma: Current understanding of the underpinning biology and clinical impact. Blood.

[4] Dimopoulos M.A., Beksac M., Pour L., Delimpasi S., Vorobyev V., Quach H., Spicka I., Radocha J., Robak P., Kim K. (2024). Belantamab Mafodotin, Pomalidomide, and Dexamethasone in Multiple Myeloma. N. Engl. J. Med..

[5] Lu Q., Yang D., Li H., Niu T., Tong A. (2024). Multiple myeloma: Signaling pathways and targeted therapy. Mol. Biomed..

[6] Zhang X., Zhang H., Lan H., Wu J., Xiao Y. (2023). CAR-T cell therapy in multiple myeloma: Current limitations and potential strategies. Front. Immunol..

[7] Bisht K., Fukao T., Chiron M., Richardson P., Atanackovic D., Chini E., Chng W.J., Van De Velde H., Malavasi F. (2023). Immunomodulatory properties of CD38 antibodies and their effect on anticancer efficacy in multiple myeloma. Cancer Med..

[8] Filho J.T.D.S., Cantadori L.O., Crusoe E.d.Q., Hungria V., Maiolino A. (2025). Daratumumab-based quadruplet versus triplet induction regimens in transplant-eligible newly diagnosed multiple myeloma: A systematic review and meta-analysis. Blood Cancer J..

[9] Sonneveld P., Dimopoulos M.A., Boccadoro M., Quach H., Ho P.J., Beksac M., Hulin C., Antonioli E., Leleu X., Mangiacavalli S. (2024). Daratumumab, Bortezomib, Lenalidomide, and Dexamethasone for Multiple Myeloma. N. Engl. J. Med..

[10] Bahlis N.J., Dimopoulos M.A., White D.J., Benboubker L., Cook G., Leiba M., Ho P.J., Kim K., Takezako N., Moreau P. (2020). Daratumumab plus lenalidomide and dexamethasone in relapsed/refractory multiple myeloma: Extended follow-up of POLLUX, a randomized, open-label, phase 3 study. Leukemia.

[11] Martin T.G., Corzo K., Chiron M., van de Velde H., Abbadessa G., Campana F., Solanki M., Meng R., Lee H., Wiederschain D. (2019). Therapeutic Opportunities with Pharmacological Inhibition of CD38 with Isatuximab. Cells..

[12] Moreno L., Perez C., Zabaleta A., Manrique I., Alignani D., Ajona D., Blanco L., Lasa M., Maiso P., Rodriguez I. (2019). The Mechanism of Action of the Anti-CD38 Monoclonal Antibody Isatuximab in Multiple Myeloma. Clin. Cancer Res..

[13] Dimopoulos M., Bringhen S., Anttila P., Capra M., Cavo M., Cole C., Gasparetto C., Hungria V., Jenner M., Vorobyev V. (2021). Isatuximab as monotherapy and combined with dexamethasone in patients with relapsed/refractory multiple myeloma. Blood.

[14] Frampton J.E. (2021). Isatuximab: A Review of Its Use in Multiple Myeloma. Target. Oncol..

[15] Rajan A.M., Kumar S. (2016). New investigational drugs with single-agent activity in multiple myeloma. Blood Cancer J..

[16] Richardson P.G., Perrot A., Miguel J.S., Beksac M., Spicka I., Leleu X., Schjesvold F., Moreau P., Dimopoulos M.A., Huang S.-Y. (2024). Isatuximab-pomalidomide-dexamethasone versus pomalidomide-dexamethasone in patients with relapsed and refractory multiple myeloma: Final overall survival analysis. Haematologica..

[17] Moreau P., Dimopoulos M.-A., Mikhael J., Yong K., Capra M., Facon T., Hajek R., Spicka I., Baker R., Kim K. (2021). Isatuximab, carfilzomib, and dexamethasone in relapsed multiple myeloma (IKEMA): A multicentre, open-label, randomised phase 3 trial. Lancet..

[18] Yong K., Martin T., Dimopoulos M.-A., Mikhael J., Capra M., Facon T., Hajek R., Spicka I., Baker R., Kim K. (2024). Isatuximab plus carfilzomib-dexamethasone versus carfilzomib-dexamethasone in patients with relapsed multiple myeloma (IKEMA): Overall survival analysis of a phase 3, randomised, controlled trial. Lancet Haematol..

[19] Goldschmidt H., Mai E.K., Bertsch U., Fenk R., Nievergall E., Tichy D., Besemer B., Durig J., Schroers R., von Metzler I. (2022). Addition of isatuximab to lenalidomide, bortezomib, and dexamethasone as induction therapy for newly diagnosed, transplantation-eligible patients with multiple myeloma (GMMG-HD7): Part 1 of an open-label, multicentre, randomised, active-controlled, phase 3 trial. Lancet Haematol..

[20] Mai E.K., Bertsch U., Pozek E., Fenk R., Besemer B., Hanoun C., Schroers R., von Metzler I., Haenel M., Mann C. (2025). Isatuximab, Lenalidomide, Bortezomib, and Dexamethasone Induction Therapy for Transplant-Eligible Newly Diagnosed Multiple Myeloma: Final Part 1 Analysis of the GMMG-HD7 Trial. J. Clin. Oncol..

[21] Facon T., Dimopoulos M.-A., Leleu X.P., Beksac M., Pour L., Hajek R., Liu Z., Minarik J., Moreau P., Romejko-Jarosinska J. (2024). Isatuximab, Bortezomib, Lenalidomide, and Dexamethasone for Multiple Myeloma. N. Engl. J. Med..

[22] Osama M., Khan M.H., Khan S., Hussain A., Tahir A., Ullah M., Afridi A., Ullah U., Rehman W.U. (2025). Efficacy and safety of anti-CD38 monoclonal antibodies-based therapy versus standard therapy in newly diagnosed multiple myeloma patients: A systematic review and meta-analysis. Ther. Adv. Hematol..

[23] Page M.J., McKenzie J.E., Bossuyt P.M., Boutron I., Hoffmann T.C., Mulrow C.D., Shamseer L., Tetzlaff J.M., Akl E.A., Brennan S.E. (2021). The PRISMA 2020 statement: An updated guideline for reporting systematic reviews. BMJ..

[24] Higgins J.P.T., Altman D.G., Gøtzsche P.C., Jüni P., Moher D., Oxman A.D., Savović J., Schulz K.F., Weeks L., Sterne J.A.C. (2011). The Cochrane Collaboration’s tool for assessing risk of bias in randomised trials. BMJ..

[25] DerSimonian R., Laird N. (2015). Meta-analysis in clinical trials revisited. Contemp. Clin. Trials.

[26] Cochran W.G. (1954). The combination of estimates from different experiments. Biometrics.

[27] Nasser M. (2020). Cochrane Handbook for Systematic Reviews of Interventions. Am. J. Public Health.

[28] Sterne J.A.C., Sutton A.J., Ioannidis J.P.A., Terrin N., Jones D.R., Lau J., Carpenter J., Rücker G., Harbord R.M., Schmid C.H. (2011). Recommendations for examining and interpreting funnel plot asymmetry in meta-analyses of randomised controlled trials. BMJ..

[29] Gay F., Roeloffzen W., Dimopoulos M.A., Rosiñol L., van der Klift M., Mina R., Oriol Rocafiguera A., Katodritou E., Wu K.L., Rodriguez Otero P. (2023). Results of the Phase III Randomized Iskia Trial: Isatuximab-Carfilzomib-Lenalidomide-Dexamethasone vs. Carfilzomib-Lenalidomide-Dexamethasone As Pre-Transplant Induction and Post-Transplant Consolidation in Newly Diagnosed Multiple Myeloma Patients. Blood.

[30] Horenstein A.L., Faini A.C., Morandi F., Ortolan E., Storti P., Giuliani N., Richardson P.G., Malavasi F. (2025). Monoclonal anti-CD38 therapy in human myeloma: Retrospects and prospects. Front. Immunol..

[31] Facon T., Moreau P., Weisel K., Goldschmidt H., Usmani S.Z., Chari A., Plesner T., Orlowski R.Z., Bahlis N., Basu S. (2025). Daratumumab/lenalidomide/dexamethasone in transplant-ineligible newly diagnosed myeloma: MAIA long-term outcomes. Leukemia.

[32] Dimopoulos M.A., Oriol A., Nahi H., San-Miguel J., Bahlis N.J., Usmani S.Z., Rabin N., Orlowski R.Z., Suzuki K., Plesner T. (2023). Overall Survival With Daratumumab, Lenalidomide, and Dexamethasone in Previously Treated Multiple Myeloma (POLLUX): A Randomized, Open-Label, Phase III Trial. J. Clin. Oncol..

[33] Zhu L., Nawaz M.A., Zuo Y., Zeng P. (2025). Next-generation immunotherapy in relapsed/refractory multiple myeloma: Strategies to achieve sustained MRD negativity. Crit. Rev. Oncol. Hematol..

[34] Kumar S., Paiva B., Anderson K.C., Durie B., Landgren O., Moreau P., Munshi N., Lonial S., Bladé J., Mateos M.-V. (2016). International Myeloma Working Group consensus criteria for response and minimal residual disease assessment in multiple myeloma. Lancet Oncol..

[35] Flores-Montero J., Sanoja-Flores L., Paiva B., Puig N., Garcia-Sanchez O., Böttcher S., van der Velden V.H.J., Pérez-Morán J.J., Vidriales M.B., García-Sanz R. (2017). Next Generation Flow for highly sensitive and standardized detection of minimal residual disease in multiple myeloma. Leukemia.

[36] Richardson P.G., O’Donnell E.K., O’Gorman P., Leypoldt L.B., Laubach J., Gay F., Leleu X., Facon T., Moreau P., Dimopoulos M.A. (2025). Isatuximab for the treatment of multiple myeloma: Current clinical advances and future directions. Expert Opin. Investig. Drugs.

[37] Lin Z., Dong R., Zhang W., Liu R., Fu B., He A. (2025). Efficacy and safety of anti-CD38 monoclonal antibodies in patients with newly diagnosed multiple myeloma: An updated systematic review and meta-analysis based on randomized controlled trials. Leuk. Lymphoma.

[38] Karimbanathottathil M.F., Yoosuf B.T., Mamatha M., Bansal D. (2024). Comprehensive safety evaluation of isatuximab in multiple myeloma using disproportionality analysis of FAERS and meta-analysis of randomized controlled trials. Sci. Rep..

[39] Quach H., Parmar G., Mateos M.V., Ailawadhi S., Leleu X. (2024). Recent Developments in Convenience of Administration of the Anti-CD38 Antibody Isatuximab: Subcutaneous Delivery and Fast Intravenous Infusion in Patients With Multiple Myeloma. Clin. Lymphoma Myeloma Leuk..

[40] Bonello F., Grasso M., D’Agostino M., Celeghini I., Castellino A., Boccadoro M., Bringhen S. (2020). The Role of Monoclonal Antibodies in the First-Line Treatment of Transplant-Ineligible Patients with Newly Diagnosed Multiple Myeloma. Pharmaceuticals.

[41] Chen W., You J., Lin L., Yan X., An G., Wang Y., Tian W., Ding K., Zhang X., Chen W. (2025). Real-world outcomes of isatuximab with pomalidomide and dexamethasone for relapsed and/or refractory multiple myeloma. Chin. Med. J. (Engl.).

