# Supplementary material for: Efficacy and Safety of Isatuximab Combination Therapy in Multiple Myeloma: A Meta-Analysis of Randomized Controlled Trials
Source: Cancers (Basel). 2025 Oct 30;17(21):3494. doi: 10.3390/cancers17213494 (PMC12606759; doi:10.3390/cancers17213494)
Supplement: Supplementary file 1 [file cancers-17-03494-s001.zip › cancers-3940764-supplementary.pdf]

Supplementary Figures.

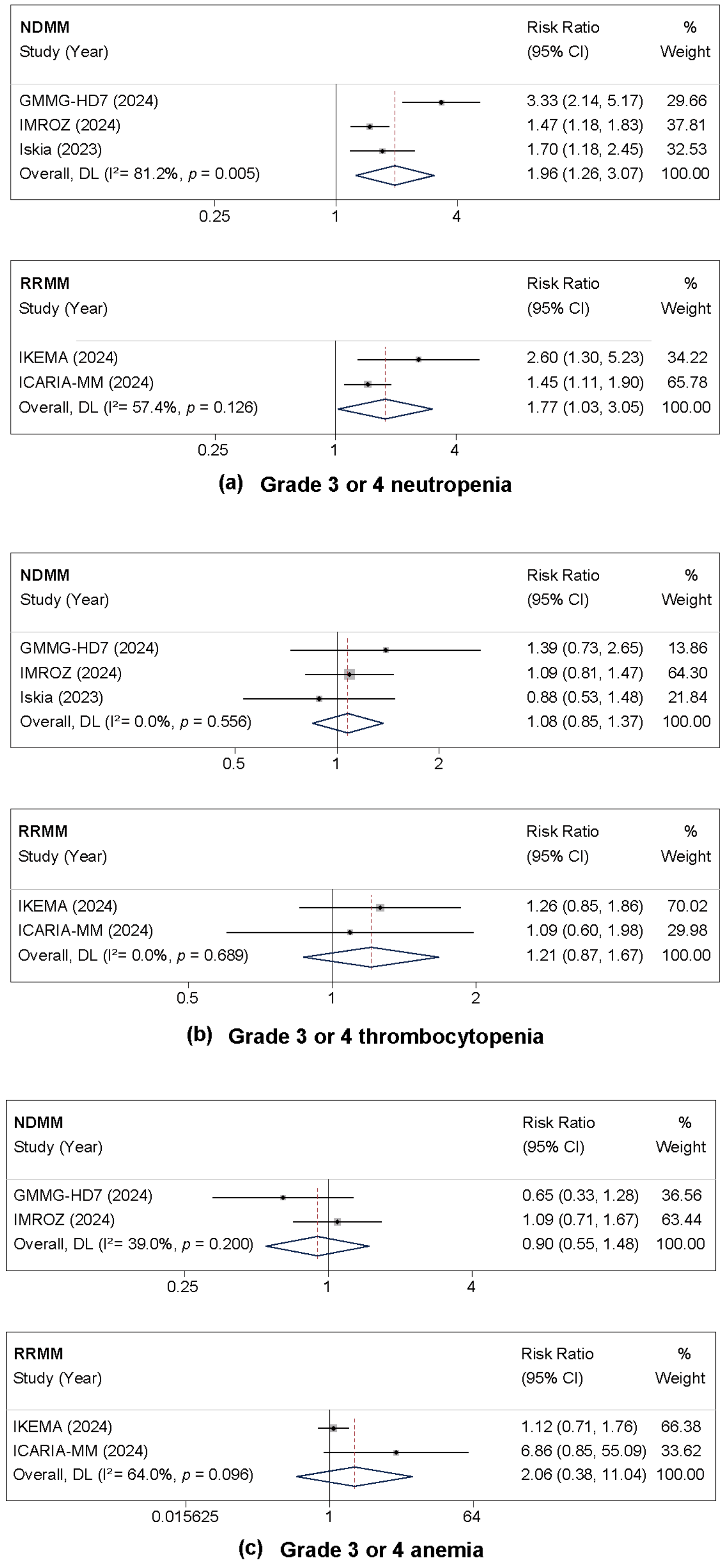

Supplementary Figure S1: Forest plots of risk ratios (RRs) for hematologic adverse events comparing isatuximab-based regimens to the control arm. (**a**) Grade 3 or 4 neutropenia. (**b**) Grade 3 or 4 thrombocytopenia. (**c**) Grade 3 or 4 anemia. For each outcome, the forest plot for

patients with newly diagnosed multiple myeloma (NDMM) is shown above the plot for those with relapsed/refractory disease (RRMM). All analyses used the random-effects model [1-6].

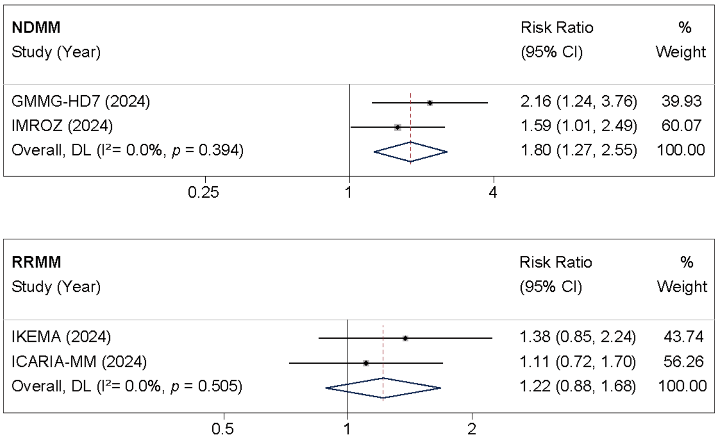

(a) Grade 3 or 4 pneumonia

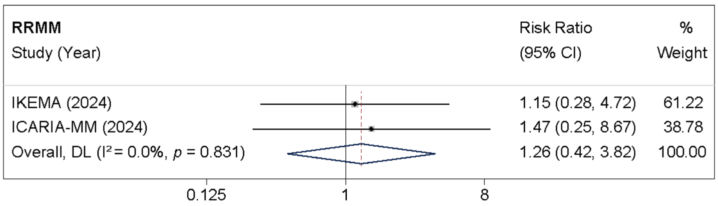

(b) Grade 3 or 4 diarrhea

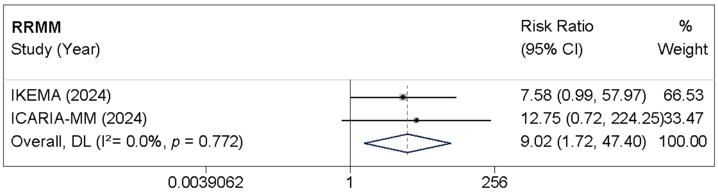

(c) Grade 3 or 4 fatigue

Supplementary Figure S2. Forest plots of risk ratios (RRs) for non-hematologic adverse events. (a) Grade 3 or 4 pneumonia (stratified by NDMM and RRMM). (b) Grade 3 or 4 diarrhea (RRMM only). (c) Grade 3 or 4 fatigue (RRMM only). Outcomes (b) and (c) are displayed for the RRMM population only due to insufficient data from NDMM studies. All analyses used the random-effects model [1-5].

Supplementary Tables.

Supplementary Table S1. PRISMA Checklist.

Section and Topic	Item #	Checklist item	Location where item is reported
**TITLE**			
Title	1	Identify the report as a systematic review.	Page 1, Line 1-3
**ABSTRACT**			
Abstract	2	See the PRISMA 2020 for Abstracts checklist.	Page 1, Abstract section (Lines 10-34)
**INTRODUCTION**			
Rationale	3	Describe the rationale for the review in the context of existing knowledge.	Introduction, Paragraphs 1-3
Objectives	4	Provide an explicit statement of the objective(s) or question(s) the review addresses.	Introduction, final paragraph
**METHODS**			
Eligibility criteria	5	Specify the inclusion and exclusion criteria for the review and how studies were grouped for the syntheses.	Section 2.2
Information sources	6	Specify all databases, registers, websites, organisations, reference lists and other sources searched or consulted to identify studies. Specify the date when each source was last searched or consulted.	Section 2.1
**Section and Topic**	**Item #**	**Checklist item**	**Location where item is reported**
Search strategy	7	Present the full search strategies for all databases, registers and websites, including any filters and limits used.	Section 2.1, Supplementary Table 1
Selection process	8	Specify the methods used to decide whether a study met the inclusion criteria of the review, including how many reviewers screened each record and each report retrieved, whether they worked independently, and if applicable, details of automation tools used in the process.	Section 2.1

Data collection process	9	Specify the methods used to collect data from reports, including how many reviewers collected data from each report, whether they worked independently, any processes for obtaining or confirming data from study investigators, and if applicable, details of automation tools used in the process.	Section 2.3
Data items	10a	List and define all outcomes for which data were sought. Specify whether all results that were compatible with each outcome domain in each study were sought (e.g. for all measures, time points, analyses), and if not, the methods used to decide which results to collect.	Abstract (Methods), Section 2.2, Section 2.3
	10b	List and define all other variables for which data were sought (e.g. participant and intervention characteristics, funding sources). Describe any assumptions made about any missing or unclear information.	Section 2.3
Study risk of bias assessment	11	Specify the methods used to assess risk of bias in the included studies, including details of the tool(s) used, how many reviewers assessed each study and whether they worked independently, and if applicable, details of automation tools used in the process.	Section 2.3 (Cochrane Risk of Bias tool)
Effect measures	12	Specify for each outcome the effect measure(s) (e.g. risk ratio, mean difference) used in the synthesis or presentation of results.	Section 2.4 (HR, RR)
Synthesis methods	13a	Describe the processes used to decide which studies were eligible for each synthesis (e.g. tabulating the study intervention characteristics and comparing against the planned groups for each synthesis (item #5)).	Section 2.4, Results 3.1
	13b	Describe any methods required to prepare the data for presentation or synthesis, such as handling of missing summary statistics, or data conversions.	Section 2.4
	13c	Describe any methods used to tabulate or visually display results of individual studies and syntheses.	Section 2.4, Figures 1-5, Table 1
	13d	Describe any methods used to synthesize results and provide a rationale for the choice(s). If meta-analysis was performed, describe the model(s), method(s) to identify the presence and extent of statistical heterogeneity, and software package(s) used.	Section 2.4 (Fixed/Random effects, I^2, RevMan/Stata)
	13e	Describe any methods used to explore possible causes of heterogeneity among study results (e.g. subgroup analysis, meta-regression).	Section 2.4 (Pre-specified subgroup analysis)
	13f	Describe any sensitivity analyses conducted to assess robustness of the synthesized results.	Section 2.4 (Not performed due to

Section and Topic	Item #	Checklist item	Location where item is reported
			limited number of studies)
Reporting bias assessment	14	Describe any methods used to assess risk of bias due to missing results in a synthesis (arising from reporting biases).	Section 2.4 (Not formally assessed due to few studies)
Certainty assessment	15	Describe any methods used to assess certainty (or confidence) in the body of evidence for an outcome.	Not reported
**RESULTS**			
Study selection	16a	Describe the results of the search and selection process, from the number of records identified in the search to the number of studies included in the review, ideally using a flow diagram.	Section 3.1, Figure 1
	16b	Cite studies that might appear to meet the inclusion criteria, but which were excluded, and explain why they were excluded.	Section 3.1, Figure 1
Study characteristics	17	Cite each included study and present its characteristics.	Section 3.1, Table 1
Risk of bias in studies	18	Present assessments of risk of bias for each included study.	Section 3.5, Figure 6
Results of individual studies	19	For all outcomes, present, for each study: (a) summary statistics for each group (where appropriate) and (b) an effect estimate and its precision (e.g. confidence/credible interval), ideally using structured tables or plots.	Figures 2, 3, 4
Results of syntheses	20a	For each synthesis, briefly summarise the characteristics and risk of bias among contributing studies.	Sections 3.2, 3.3, 3.5
	20b	Present results of all statistical syntheses conducted. If meta-analysis was done, present for each the summary estimate and its precision (e.g. confidence/credible interval) and measures of statistical heterogeneity. If comparing groups, describe the direction of the effect.	Sections 3.2, 3.3, Figures 2-4
	20c	Present results of all investigations of possible causes of heterogeneity among study results.	Section 3.4, Section 3.2.2
	20d	Present results of all sensitivity analyses conducted to assess the robustness of the synthesized results.	Section 2.4 states none were performed

Reporting biases	21	Present assessments of risk of bias due to missing results (arising from reporting biases) for each synthesis assessed.	As per Methods (Item 14), not assessed
Certainty of evidence	22	Present assessments of certainty (or confidence) in the body of evidence for each outcome assessed.	Not reported
**DISCUSSION**			
Discussion	23a	Provide a general interpretation of the results in the context of other evidence.	Discussion, Paragraphs 1-5
	23b	Discuss any limitations of the evidence included in the review.	Discussion, Paragraph 6
	23c	Discuss any limitations of the review processes used.	Discussion, Paragraph 6
	23d	Discuss implications of the results for practice, policy, and future research.	Discussion, Paragraph 5 & 7, Conclusion
**OTHER INFORMATION**			
**Section and Topic**	**Item #**	**Checklist item**	**Location where item is reported**
Registration and protocol	24a	Provide registration information for the review, including register name and registration number, or state that the review was not registered.	Section 2.1: "The study was registered in the International Platform of Registered Systematic Review and Meta-analysis Protocols (registration number: INPLASY2025.10.0027)."
	24b	Indicate where the review protocol can be accessed, or state that a protocol was not prepared.	A protocol was prepared and registered (DOI:

			10.37766/inplasy2025.10.0027).
	24c	Describe and explain any amendments to information provided at registration or in the protocol.	Not applicable
Support	25	Describe sources of financial or non-financial support for the review, and the role of the funders or sponsors in the review.	Funding section
Competing interests	26	Declare any competing interests of review authors.	Conflicts of Interest section
Availability of data, code and other materials	27	Report which of the following are publicly available and where they can be found: template data collection forms; data extracted from included studies; data used for all analyses; analytic code; any other materials used in the review.	Data Availability Statement, Supplementary Materials

Supplementary Table S2. Search strategy.

Search engine	Search string	Search result
**PubMed**	("isatuximab"[Supplementary Concept] OR ("SAR650984"[Title/Abstract] OR "Sarclisa"[Title/Abstract] OR "isatuximab-irfc"[Title/Abstract])) AND ("Multiple Myeloma"[MeSH Terms] OR ("myeloma plasma cell"[Title/Abstract] OR "plasma cell myeloma"[Title/Abstract] OR "kahler disease"[Title/Abstract] OR (("Disease"[MeSH Terms] OR "Disease"[All Fields] OR "diseases"[All Fields] OR "diseases"[All Fields] OR "diseased"[All Fields]) AND "Kahler"[Title/Abstract]) OR "Myelomatosis"[Title/Abstract])) AND "Randomized Controlled Trial"[Publication Type]	**28**

Supplementary Table S3. Indirect Comparison of Key Efficacy and Safety Outcomes for Anti-CD38 Antibodies in Multiple Myeloma.

Outcome	Population	Isatuximab-based Therapy (Data from this meta-analysis)	Daratumumab-based Therapy (Data from key published trials)
PFS (HR)	NDMM	0.66 (0.52-0.84) [Pooled from IMROZ, GMMG-HD7]	0.55 (0.45-0.67) [MAIA: D-Rd vs Rd]
	RRMM	0.61 (0.50-0.74) [Pooled from IKEMA, ICARIA-MM]	0.54 (0.43-0.67) [POLLUX: D-Rd vs Rd]
OS (HR)	NDMM	1.01 (0.72-1.43) [Pooled from IMROZ, GMMG-HD7]	0.66 (0.53-0.83) [MAIA: D-Rd vs Rd]
	RRMM	0.81 (0.65-1.00) [Pooled from IKEMA, ICARIA-MM]	0.73 (0.58-0.91) [POLLUX: D-Rd vs Rd]
MRD negative rate	NDMM	1.28 (1.13-1.45) [Pooled from IMROZ, GMMG-HD7, IsKia]	32.1% vs 11.1% [MAIA: D-Rd vs Rd]
	RRMM	4.37 (0.60-31.68)	33.2% vs 6.7%

		[Pooled from IKEMA, ICARIA-MM]	[POLLUX: D-Rd vs Rd]
Grade 3/4 neutropenia	NDMM	1.96 (1.26-3.07)<br>[Pooled from GMMG-HD7, IMROZ, IsKia]	54.1% vs 37.0%<br>[MAIA: D-Rd vs Rd]
	RRMM	1.77 (1.03-3.05)<br>[Pooled from IKEMA, ICARIA-MM]	57.6% vs 41.6%<br>[POLLUX: D-Rd vs Rd]
Grade 3/4 anemia	NDMM	0.90 (0.55-1.48)<br>[Pooled from GMMG-HD7, IMROZ]	17.0% vs 21.6%<br>[MAIA: D-Rd vs Rd]
	RRMM	2.06 (0.39-11.04)<br>[Pooled from IKEMA, ICARIA-MM]	19.8% vs 22.4%<br>[POLLUX: D-Rd vs Rd]
Grade 3/4 pneumonia	NDMM	1.80 (1.27-2.55)<br>[Pooled from GMMG-HD7, IMROZ]	19.5% vs 10.7%<br>[MAIA: D-Rd vs Rd]
	RRMM	1.22 (0.88-1.68)<br>[Pooled from IKEMA, ICARIA-MM]	17.3% vs 11.0%<br>[POLLUX: D-Rd vs Rd]

This table provides a descriptive, indirect comparison of outcomes for isatuximab and daratumumab from separate clinical trial populations. Data sources and formats differ between columns: Efficacy and safety data for isatuximab are pooled estimates (Hazard Ratios, HR, or Risk Ratios, RR with 95% CI) from the current meta-analysis of randomized controlled trials. Data for daratumumab are selected results from key pivotal trials (MAIA for NDMM, POLLUX for RRMM), presented as reported in the primary literature (HRs for time-to-event outcomes; incidence rates for safety outcomes). These data originate from different trials with varying designs, patient characteristics, and control arms. Therefore, direct statistical comparison of the values between columns is not methodologically valid and should be avoided. The table aims to contextualize the findings of this meta-analysis within the broader landscape of anti-CD38 therapies. CI, confidence interval; NDMM, newly diagnosed multiple myeloma; RRMM, relapsed/refractory multiple myeloma [1–8].

References

1. Richardson, P.G.; Perrot, A.; Miguel, J.S.; Beksac, M.; Spicka, I.; Leleu, X.; Schjesvold, F.; Moreau, P.; Dimopoulos, M.A.; Huang, S.-Y.; et al. Isatuximab-pomalidomide-dexamethasone versus pomalidomide-dexamethasone in patients with relapsed and refractory multiple myeloma: final overall survival analysis. *Haematologica.* **2024**, *109*, 2239–2249. https://doi.org/10.3324/haematol.2023.284325.
2. Yong, K.; Martin, T.; Dimopoulos, M.-A.; Mikhael, J.; Capra, M.; Facon, T.; Hajek, R.; Spicka, I.; Baker, R.; Kim, K.; et al. Isatuximab plus carfilzomib-dexamethasone versus

carfilzomib-dexamethasone in patients with relapsed multiple myeloma (IKEMA): overall survival analysis of a phase 3, randomised, controlled trial. *Lancet Haematol.* **2024**, *11*, e741–e750. https://doi.org/10.1016/S2352-3026(24)00148-0.
3. Goldschmidt, H.; Mai, E.K.; Bertsch, U.; Fenk, R.; Nievergall, E.; Tichy, D.; Besemer, B.; Durig, J.; Schroers, R.; von Metzler, I.; et al. Addition of isatuximab to lenalidomide, bortezomib, and dexamethasone as induction therapy for newly diagnosed, transplantation-eligible patients with multiple myeloma (GMMG-HD7): part 1 of an open-label, multicentre, randomised, active-controlled, phase 3 trial. *Lancet Haematol.* **2022**, *9*, e810–e821. https://doi.org/10.1016/S2352-3026(22)00263-0.
4. Mai, E.K.; Bertsch, U.; Pozek, E.; Fenk, R.; Besemer, B.; Hanoun, C.; Schroers, R.; von Metzler, I.; Haenel, M.; Mann, C.; et al. Isatuximab, Lenalidomide, Bortezomib, and Dexamethasone Induction Therapy for Transplant-Eligible Newly Diagnosed Multiple Myeloma: Final Part 1 Analysis of the GMMG-HD7 Trial. *J. Clin. Oncol.* **2025**, *43*, 1279–1288. https://doi.org/10.1200/JCO-24-02266.
5. Facon, T.; Dimopoulos, M.-A.; Leleu, X.P.; Beksac, M.; Pour, L.; Hajek, R.; Liu, Z.; Minarik, J.; Moreau, P.; Romejko-Jarosinska, J.; et al. Isatuximab, Bortezomib, Lenalidomide, and Dexamethasone for Multiple Myeloma. *N. Engl. J. Med.* **2024**, *391*, 1597–1609. https://doi.org/10.1056/NEJMoa2400712.
6. Gay, F.; Roeloffzen, W.; Dimopoulos, M.A.; Rosiñol, L.; van der Klift, M.; Mina, R.; Oriol Rocafiguera, A.; Katodritou, E.; Wu, K.L.; Rodriguez Otero, P.; et al. Results of the Phase III Randomized Iskia Trial: Isatuximab-Carfilzomib-Lenalidomide-Dexamethasone vs. Carfilzomib-Lenalidomide-Dexamethasone As Pre-Transplant Induction and Post-Transplant Consolidation in Newly Diagnosed Multiple Myeloma Patients. *Blood* **2023**, *142*, 4. https://doi.org/10.1182/blood-2023-177546.
7. Facon, T.; Moreau, P.; Weisel, K.; Goldschmidt, H.; Usmani, S.Z.; Chari, A.; Plesner, T.; Orlowski, R.Z.; Bahlis, N.; Basu, S.; et al. Daratumumab/lenalidomide/dexamethasone in transplant-ineligible newly diagnosed myeloma: MAIA long-term outcomes. *Leukemia* **2025**, *39*, 942–950. https://doi.org/10.1038/s41375-024-02505-2.
8. Dimopoulos, M.A.; Oriol, A.; Nahi, H.; San-Miguel, J.; Bahlis, N.J.; Usmani, S.Z.; Rabin, N.; Orlowski, R.Z.; Suzuki, K.; Plesner, T.; et al. Overall Survival With Daratumumab, Lenalidomide, and Dexamethasone in Previously Treated Multiple Myeloma (POLLUX): A Randomized, Open-Label, Phase III Trial. *J. Clin. Oncol.* **2023**, *41*, 1590–1599. https://doi.org/10.1200/JCO.22.00940
